# Photon-counting detector computed tomography: iodine density versus virtual monoenergetic imaging of pancreatic ductal adenocarcinoma

**DOI:** 10.1007/s00261-024-04605-0

**Published:** 2024-09-26

**Authors:** Zlatan Alagic, Carlos Valls Duran, Chikako Suzuki, Kolbeinn Halldorsson, Anders Svensson-Marcial, Rebecca Saeter, Seppo K. Koskinen

**Affiliations:** 1 Department of Diagnostic Radiology, Karolinska University Hospital, Stockholm, 171 76 Sweden; 2 Department of Clinical Science, Intervention and Technology (CLINTEC), Karolinska Institutet, Stockholm, 171 77 Sweden; 3 Department of Molecular Medicine and Surgery, Karolinska Institutet, Stockholm, 171 77 Sweden; 4 Department of Diagnostic Radiology, Stockholm South General Hospital, Stockholm, 118 83 Sweden; 5 Department of Medical Physics and Nuclear Medicine, Karolinska University Hospital, Stockholm, 171 76 Sweden

## Introduction

Pancreatic carcinoma has a poor prognosis and is the seventh leading cause of cancer-associated deaths worldwide [1]. The most common primary pancreatic carcinoma is pancreatic ductal adenocarcinoma (PDAC) [2]. Computed tomography (CT) is the workhorse for the evaluation of local invasion of pancreatic tumors and assessment of resectability [3]. A typical CT pancreas protocol is a dual phase acquisition comprising a late arterial phase (LAP), also known as pancreatic phase, 35–40 s after contrast injection and a portal venous phase (PVP) at around 70 s [4]. The LAP offers a greater attenuation difference between the enhancing pancreatic parenchyma and the hypoattenuating PDAC [5]. However, a LAP is not always available, such as during an initial CT scan of the patient or during a follow-up CT in patients with metastatic disease when the examination is performed only in the PVP.

Recently a first-generation dual-source photon-counting detector computed tomography (PCD-CT) scanner became clinically available [6]. Unlike energy-integrating detector CT (EID-CT), which down-weights low-energy photons, PCD-CT counts photons equally across the entire X-ray energy spectrum, thereby increasing the iodine signal. Moreover, since the energy information of human organs primarily resides within the relatively low-energy X-ray spectrum, PCD-CT improves soft tissues contrast. Setting the low energy threshold slightly above electronic noise levels effectively minimizes noise. Additionally, PCD-CT’s smaller detector pixel size, compared to EID-CT, enhances spatial resolution. Combined with improved iodine contrast, this can enhance the detectability of small objects [7, 8]. This may be particularly significant for detecting PDAC in the PVP, where the attenuation differences between pancreatic parenchyma and the tumor are less pronounced compared to the LAP [5]. Furthermore, PCD-CT obtains spectral information from every scan because the energy of each detected photon is registered and binned. This energy information can be utilized to generate virtual monoenergetic images (VMIs), and for material decomposition, such as generating material density iodine (MD-iodine) images [9]. Previous single-energy EID-CT studies have demonstrated that to increase the contrast between pancreatic parenchyma and tumors, a low tube voltage can be utilized. This approach narrows the difference between the mean energy of the x-ray spectrum and the iodine k-edge (33.2 keV), resulting in enhanced iodine x-ray absorption [10, 11]. Moreover, previous DECT-studies [12–24] and recent PCD-CT studies [25, 26] have concluded that pancreatic carcinoma is better depicted on low-energy VMIs. However, image noise increases at lower keV levels, which decreases the overall image quality [13, 14, 23–26]. The current use of MD-iodine images in abdominal dual-energy CT (DECT) has primarily relied on iodine concentration measurements. The use of MD-iodine images for diagnosis has been hindered by high image noise and low spatial resolution [27]. Only a few spectral CT studies, all DECT, have investigated MD-iodine imaging for the assessment of PDAC, and the findings have shown some variability across different contrast phases. Two previous DECT-studies in LAP have shown that MD-iodine images exhibit significantly higher contrast-to-noise ratio (CNR) for PDAC compared to 70 keV and low keV-images [14, 28]. Additionally, MD-iodine images received the highest reader scores for lesion conspicuity [14]. Conversely, a DECT-study in PVP reported no significant difference in lesion conspicuity between MD-iodine and 40 keV VMI [29]. The potential of MD-iodine images to further improve the assessment of PDAC with PCD-CT has not yet been evaluated. Therefore, the aim of this study was to conduct a quantitative and qualitative analysis of the image quality of PCD-CT for PDAC on MD-iodine images compared to VMIs at different keV levels in the PVP.

## Methods

The regional ethics committee has approved this retrospective study (Dnr 2023-01724-01) and informed consent was waived due to the retrospective design.

### Patient inclusion and clinical data

**Fig. 1:**
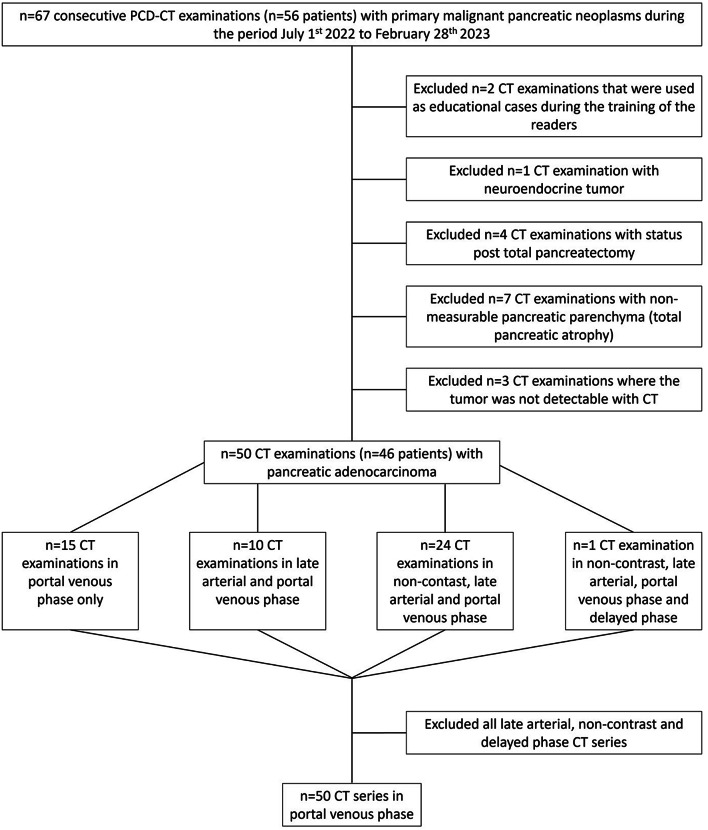
Flowchart of patient inclusion

We retrospectively included 56 consecutive patients (67 CT examinations) with primary malignant pancreatic neoplasms that were scanned on a first-generation dual-source PCD-CT (NAEOTOM Alpha, Siemens Healthineers, Forchheim, Germany) between the period 1 July 2022 to 28 February 2023 at a tertiary referral center at Karolinska University Hospital, Stockholm, Sweden. 17 PCD-CT examinations were excluded: 2 examinations that were used for educational purposes during reader training, 1 due to a neuroendocrine tumor, 4 because of total pancreatectomy, 7 with non-measurable pancreatic parenchyma due to total atrophy, and 3 because the tumor was not detectable with CT. Hence, a total of 50 PCD-CT examinations (46 patients) with PDAC were included in the study. The patient inclusion is depicted in Fig. 1.

Collected clinical data included sex, age, BMI, histopathological reports, and ongoing neoadjuvant or adjuvant therapy. Maximum tumor diameters were measured on axial images on the 70 keV VMI.

PDAC was confirmed by cytology or histology in all cases except in one case where an elderly patient with several comorbidities had a locally advanced pancreatic tumor with dissemination and where palliative care was decided. CT in this case showed typical findings of PDAC, and the patient also had elevated tumor markers (CA 19-9, CA 125, and CEA).

### PCD-CT protocol and image reconstruction

The abdominal scans were acquired on a dual-source PCD-CT (NAEOTOM Alpha, Siemens Healthineers, Forchheim, Germany), employing various acquisition protocols: monophasic in PVP (70 s after contrast injection); biphasic with scanning in LAP (35–40 s after contrast injection) followed by PVP; triphasic, which is our CT pancreas protocol, including a non-contrast phase in addition to LAP and PVP; and quadriphasic, which includes a late phase (160 s after contrast injection), in addition to non-contrast phase, LAP and PVP. 15 of the PCD-CT examinations were monophasic, 10 were biphasic, 24 were triphasic, and one was quadriphasic (Fig. 1). All patients scanned with the monophasic acquisition protocol had confirmed or suspected intra-abdominal metastases according to their imaging referrals. Of the 10 patients scanned with the biphasic protocol, 6 had previously confirmed intra-abdominal metastases. Among the 24 patients scanned with the triphasic protocol, 2 had previously confirmed intra-abdominal metastases. The one patient that was scanned with the quadriphasic protocol had suspected liver metastases that were difficult to differentiate from liver abscesses. Thus, the triphasic acquisition (CT pancreas protocol) was primarily reserved for patients with localized disease and no metastasis.

The contrast agent iodixanol (Visipaque^®^ 320 mgI/ml, GE Healthcare, Princeton, NJ, USA) was administered through a peripheral vein in the forearm with a dose of 0.5 g of iodine per kg of body weight (maximum dosage weight 100 kg for men and 80 kg for women) with a fixed injection time of 25 s.

The following imaging parameters were applied: multi-energy acquisition mode (QuantumPlus, Siemens Healthineers); automatic exposure control with axial and longitudinal tube current modulation (CARE Dose4D, Siemens Healthineers) with an IQ level of 304; tube potential, 140 kV fixed; rotation time, 0.5 s; pitch, 0.8; collimation, 144 × 0.4 mm; kernel, Br44; iterative reconstruction, QIR 3. The QuantumPlus mode cannot currently be combined with the ultra-high resolution mode (collimation, 120 × 0.2 mm) on the PCD-CT. Nevertheless, the QuantumPlus mode still provides high spatial resolution with a minimum section thickness of 0.4 mm. From the spectral data, MD-iodine images were generated, as well as VMI reconstructions at 55 keV and 70 keV, for all contrast phases.

All CT series were reconstructed with 3 mm slice thickness and 1.5 mm increment, which is the standard in our routine clinical abdominal CT protocols.

### Quantitative image quality analysis

The CT series were quantitatively analyzed on a PACS workstation (Sectra PACS IDS7, v.23.1, Linkoping, Sweden) by a radiologist with 10 years’ CT experience (ZA) who did not participate in the qualitative analysis.

Without exceeding the size of the tumor or the normal pancreatic tissue an as large as possible circular region of interest (ROI) was placed in the tumor and a same size ROI in the normal pancreatic tissue, on three consecutive slices. Necrotic tumor areas and vessels were spared. From the ROIs, the mean HU values and the standard deviation (SD) of HU values from the three consecutive slices were averaged to calculate the CNR. The image noise was defined as the SD of HU values. The ROI size and placement was identical in all CT series.

CNR between the normal pancreatic parenchyma and the tumor was calculated by the following formula:

$$\:\text{CNR}\text{\:}\text{=}\text{\:}\frac{\left|{\text{m}\text{e}\text{a}\text{n}\:\text{H}\text{U}}_{\text{p}\text{a}\text{n}\text{c}\text{r}\text{e}\text{a}\text{s}}-{\text{m}\text{e}\text{a}\text{n}\:\text{H}\text{U}}_{\text{t}\text{u}\text{m}\text{o}\text{r}}\right|}{\sqrt{{(\text{S}\text{D}\:\text{H}\text{U}}_{\text{p}\text{a}\text{n}\text{c}\text{r}\text{e}\text{a}\text{s}}^{2}+\:{\text{S}\text{D}\:\text{H}\text{U}}_{\text{t}\text{u}\text{m}\text{o}\text{r}}^{2})\times\:0.5}}$$

To determine if BMI affects the CNR, the correlation between BMI and CNR was calculated.

### Qualitative image quality analysis

The assessment of the qualitative image quality between the different image reconstructions (MD-iodine, 55 keV VMI, and 70 keV VMI) was performed independently by three readers who are abdominal radiologists: R1 with 20 years’ experience, R2 with 20 years’ experience, and R3 with 2 years’ experience. The assessment was done on a clinical PACS workstation (Sectra PACS IDS7, v.23.1, Linkoping, Sweden). The readers were blinded to who the other readers were, to each other’s assessments, to the results from the quantitative analysis, and to the specific reconstructions of the CT series. Prior to the qualitative image quality assessment, every reader was independently trained for 1 h including a presentation of educational pancreatic carcinoma cases with associated image quality grading that was decided in advance through consensus of two experienced radiologists (SKK and ZA). The educational cases were not included in the study.

For each PCD-CT examination anonymized CT series comprising MD-iodine, 55 keV, and 70 keV were displayed in a random arrangement next to one another. The CT series comprised the same CT series that were quantitatively analyzed.

Qualitative image quality parameters comprised: “tumor conspicuity”, “sharpness of pancreatic and surrounding structures” (“structures”), “image noise”, and “overall image quality”. The grading system was constructed upon the European guidelines on quality criteria for CT [30] and comprised a five-point Likert scale (5 = best, 1 = worst) which is presented in Table 1. The tumor location was obtained from the original abdominal CT reports and included in the assessment table of each patient.

**Table 1:** Grading system for the assessment of the qualitative image quality

Parameter	Score				
	1	2	3	4	5
Lesion conspicuity	Not visualized at all, non-diagnostic	Poorly visualized	Moderately visualized	Well visualized	Very well visualized
Image noise	Very noisy, non-diagnostic	Noisy	Moderate noise	Low noise	Little to no noise
Pancreatic and surrounding structures*	Very poor sharpness, very blurry, non-diagnostic	Poor sharpness, blurry	Moderate sharpness	Good sharpness	Very good sharpness, sharpest
Overall image quality	Very poor overall image quality, non-diagnostic	Poor overall image quality	Satisfactory overall image quality	Good overall image quality	Very good overall image quality

* pancreatic contours, pancreatic parenchyma, pancreatic duct, common bile duct, mesenteric artery and vein, splenic artery and vein, portal vein, coeliac trunk, aorta, vena cava, renal vessels, duodenum

### Statistical analysis

The data analysis was performed with the statistical software IBM SPSS (v.28, Chicago, IL, USA). Ordinal variables were presented as percentages and continuous variables as mean ± SD. The two-sided significance level was set at 0.05.

The continuous variables were compared between the different image reconstructions (MD-iodine, 55 keV VMI, and 70 keV VMI) through a one-way repeated-measures analysis of variance (ANOVA), and if assumptions were not met, the Friedman test was conducted instead.

The correlation between BMI and image quality was evaluated by Spearman’s rank correlation coefficient (ρ) and interpreted as suggested by Chan [31, 32]: <⎟ 0.3 ⎜poor,⎟ 0.3–0.5 ⎜fair,⎟ 0.6–0.8 ⎜moderately strong, and ≥⎟ 0.8 ⎜very strong.

The ordinal variables from the qualitative analysis were compared between the different image reconstructions through the Friedman test.

A statistically significant repeated-measures ANOVA was followed by a post hoc pairwise comparison through the paired t-test with Bonferroni correction. A statistically significant Friedman test was followed by post hoc pairwise comparisons through the Dunn-Bonferroni test.

The Shapiro-Wilks test was used for normality testing. Validation of the repeated-measures ANOVA for sphericity was conducted through Mauchly’s sphericity test.

The inter-reader agreement was conducted through a linearly weighted kappa (κ_w_) and described according to Altman [33]: < 0.20 poor, 0.21–0.40 fair, 0.41–0.60 moderate, 0.61–0.80 good, and 0.81–1.00 very good agreement.

## Results

### Patient population and clinical characteristics

The cohort comprised 27 females and 19 males, mean age 68.9 (range 45–84), mean BMI 24.1 (range 15.2–32.1). Mean tumor size was 38.9 (range 12–88 mm). Mean ROI-diameter was 7.3 (range 5–10 mm), and mean ROI-area was 45.5 mm^2^ (range 19.6–78.5 mm^2^).

Of the included 50 PCD-CT examinations (46 patients), 10 patients (10 PCD-CT examinations) had tumor recurrence after surgery (9 Whipple procedures and 1 distal pancreatic resection) of which 3 were receiving chemotherapy. Of the remaining 40 patients that did not have surgery 21 patients (24 PCD-CT examinations) had ongoing chemotherapy.

### Comparison of quantitative image quality

**Fig. 2:**
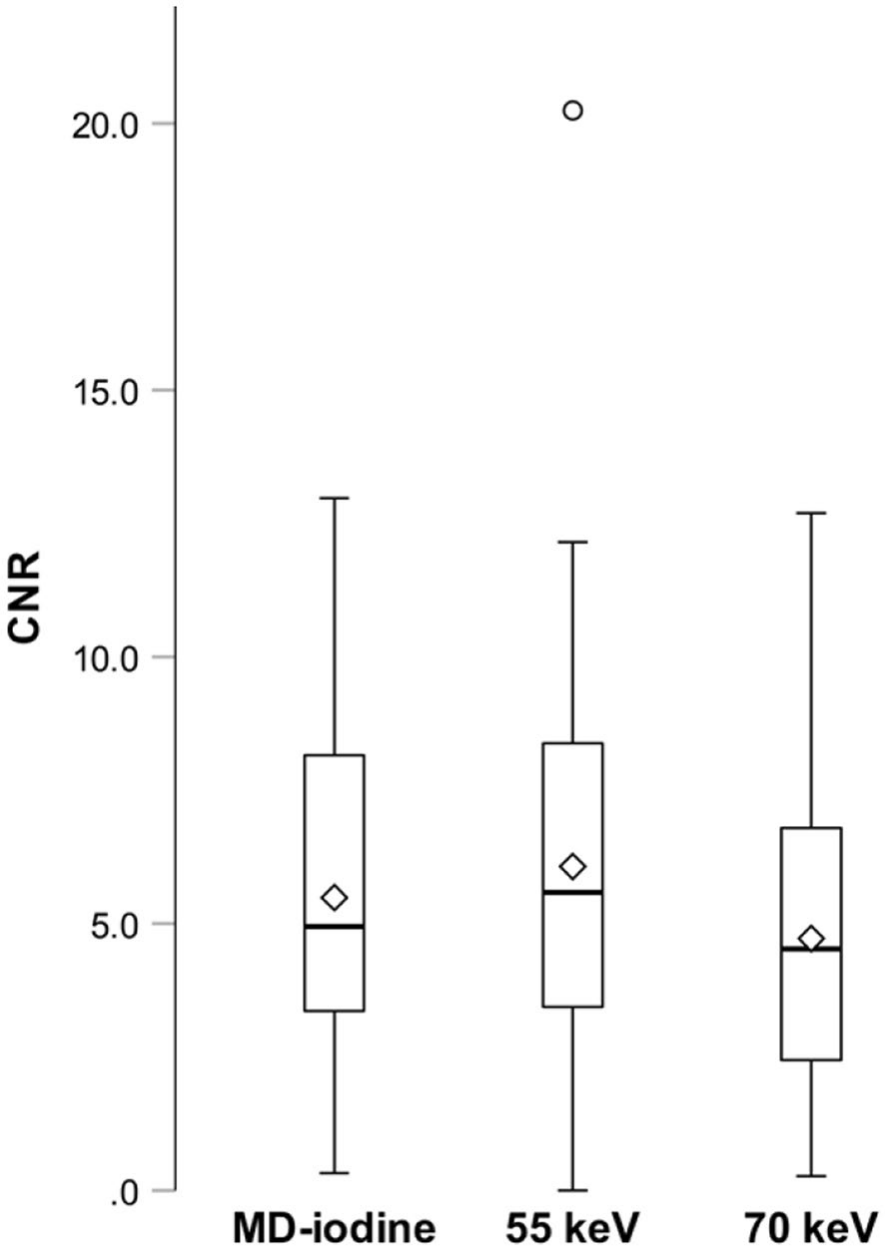
Boxplot diagram of CNR between normal pancreatic parenchyma and tumor for MD-iodine, 55 keV, and 70 keV in portal venous phase. The middle 50% of the data is represented by boxes, the median by solid lines, the mean by white diamonds, the minimum and maximum values by whiskers, and the outliers by circles

The results from the quantitative image quality analysis are summarized in Table 2, and Fig. 2.

The CNR was significantly higher on MD-iodine compared to 70 keV (*p* = 0.003). However, MD-iodine achieved significantly lower CNR compared to 55 keV (*p* = 0.049). 55 keV had significantly higher CNR than 70 keV (*p* < 0.001).

No significant correlations between BMI and CNR were detected (*p* ranging from 0.214 to 0.331).

**Table 2:** Comparison of quantitative image quality parameters between the different image reconstructions MD-iodine, 55 keV, and 70 keV, in portal venous phase

	MD-iodine	55 keV	70 keV	*p* value
CT Attenuation (HU)				
Pancreatic parenchyma	63.53 ± 20.59	150.13 ± 34.21	105.14 ± 20.17	
Tumor	22.77 ± 17.30	75.34 ± 31.48	59.12 ± 19.79	
**Image noise (HU)**				
Pancreatic parenchyma	7.85 ± 2.09	12.80 ± 3.58	9.66 ± 2.37	
Tumor	7.84 ± 2.74	13.60 ± 5.31	10.56 ± 3.43	
**CNR**				
Tumor-pancreatic parenchyma	5.49 ± 3.00^ab^	6.07 ± 3.77^b^	4.72 ± 2.79	*< 0.001*

*HU* Hounsfield Units, *CNR* contrast-to-noise ratio, *keV* kiloelectron volts, *MD-iodine* material density iodine

Post hoc pairwise multiple comparisons procedure with the Dunn-Bonferroni test showed a statistically significant (*P* < 0.05) difference between means when compared with 55 keV (^a^), and 70 keV (^b^)

### Comparison of qualitative image quality

**Fig. 3:**
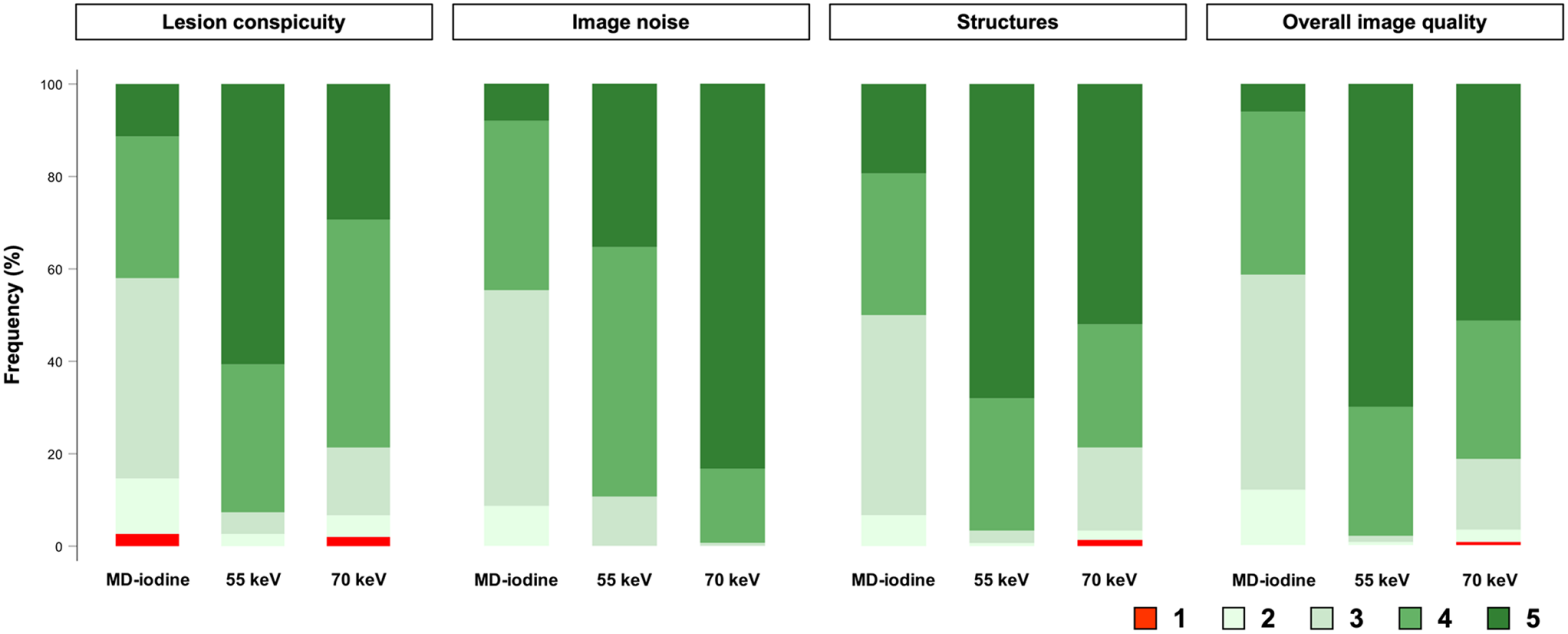
Stacked bar charts showing cumulative reader scores as percentages for MD-iodine, 55 keV, and 70 keV, for the qualitative image quality parameters (“lesion conspicuity”, “image noise”, pancreatic and surrounding structures (“structures”), and “overall image quality”)

**Fig. 4:**
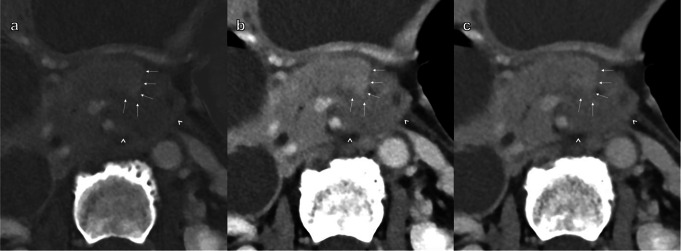
Axial contrast-enhanced abdominal CT scan in portal venous phase. Image **a** is MD-iodine, image **b** is VMI at 55 keV, and image **c** is VMI at 70 keV. The images are of a 56-year-old female patient with a PDAC in the body of the pancreas. The white arrows indicate the border between the tumor and normal pancreatic parenchyma. The arrowheads indicate the border between the tumor and the retroperitoneal fat

**Fig. 5:**
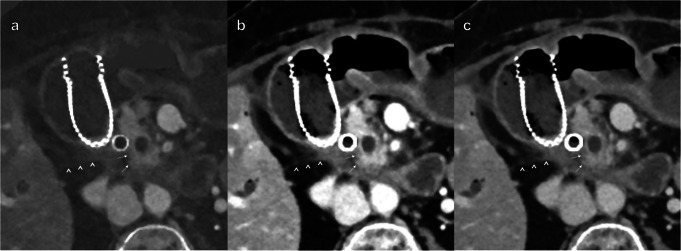
Axial contrast-enhanced abdominal CT scans in portal venous phase. Image **a** is MD-iodine, image **b** is VMI at 55 keV, and image **c** is VMI at 70 keV. The images are of a 79-year-old female patient with a PDAC in the head of the pancreas partially surrounding the biliary stent. The white arrows indicate the border between the tumor and normal pancreatic parenchyma. The arrowheads indicate tumoral strands extending laterally from the tumor into the retroperitoneal fat. One of the most experienced readers (Reader 1) graded MD-iodine in this case as Score 1 for the parameter lesion conspicuity (Lesion not visualized at all). The remaining image reconstructions were graded as Score 2

**Fig. 6:**
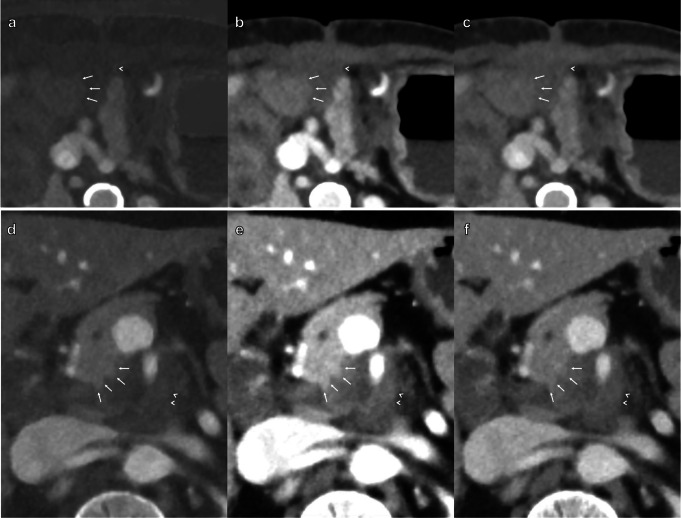
Axial contrast-enhanced abdominal CT scans in portal venous phase. The first column of images (**a** and **d**) is MD-iodine, the second column (**b** and **e**) VMI at 55 keV, and the third column (**c** and **f**) VMI at 70 keV. The first row of images (**a-c**) is of an 82-year-old female patient with a tumor recurrence post Whipple procedure at the level of the pancreaticojejunostomy. The white arrows indicate the border between the tumor and the jejunal wall. The arrowhead indicates the border between the tumor and the mesenteric fat. The second row of images (**d-f**) is of a 45-year-old male with a PDAC in the uncinate process of the pancreas encasing the superior mesenteric artery. The white arrows indicate the border between the tumor and normal pancreatic parenchyma. The arrowheads indicate the border between the tumor and the retroperitoneal fat. One of the most experienced readers (Reader 1) graded MD-iodine in the case of the first row as Score 1 for the parameter lesion conspicuity (Lesion not visualized at all), 70 keV as Score 3, and 55 keV as Score 5

The cumulative reader scores are summarized in Table 3, and in Fig. 3. The p-values from the post hoc pairwise comparisons are presented in Supplementary Table1. The scores for each specific reader are presented in Supplementary Table 2 and in Supplementary Fig. 1. MD-iodine received the lowest cumulative reader scores for all parameters (Figs. 4, 5 and 6; Table 3), and this finding was statistically significant. 55 keV received the highest scores for all parameters except “image noise”, and this finding was also statistically significant. “Image noise” was graded lowest (highest scores) on 70 keV.

R1 assigned Score 1 (non-diagnostic) for “lesion conspicuity” to four MD-iodine CT-series, with three of these also receiving Score 1 on the 70 keV reconstruction (Supplementary Table2). R2 assigned Score 1 for “structures” to two 70 keV CT-series, and for “overall image quality” to one 70 keV CT-series. R3 did not assign a Score 1 to any CT-series. There were no non-diagnostic 55 keV CT series among the readers for any parameter.

The inter-reader agreement is presented in Table 4. All κ_w_ were statistically significant, except κ_w_ for “structures” between R1 and R2. For “lesion conspicuity”, κ_w_ showed fair agreement (κ_w_ = 0.241) between R1 and R3, fair agreement (κ_w_ = 0.274) between R1 and R2, and poor (κ_w_ = 0.183) agreement between R2 and R3. For “image noise”, κ_w_ showed fair agreement (κ_w_ = 0.368) between R1 and R3, fair agreement (κ_w_ = 0.307) between R1 and R2, and fair agreement (κ_w_ = 0.338) between R2 and R3. For “structures”, significant κ_w_ showed moderate agreement (κ_w_ = 0.459) between R1 and R3, and poor agreement (κ_w_ = -0.143) between R2 and R3. For “overall image quality”, κ_w_ showed fair agreement (κ_w_ = 0.379) between R1 and R3, poor agreement (κ_w_ = 0.185) between R1 and R2, and poor agreement (κ_w_ = 0.120) between R2 and R3.

**Table 3:** Comparison of cumulative qualitative image quality scores from all readers for the abdominal portal venous phase CT series between MD-iodine, 55 keV, and 70 keV

Parameters, Subgroups (Distribution of scores 1/2/3/4/5 given as percentages)		MD-iodine	55 keV	70 keV	*p* value
	Lesion conspicuity	2.7/12.0/43.3/30.7/11.3^ab^	0/2.7/4.7/32.0/60.7^b^	2.0/4.7/14.7/49.3/29.3	*< 0.001*
	Image noise	0/8.7/46.7/36.7/8.0^ab^	0/0/10.7/54.0/35.3^b^	0/0/0.7/16.0/83.3	*< 0.001*
	Pancreatic and surrounding structures	0/6.7/43.3/30.7/19.3^ab^	0/0.7/2.7/28.7/68.0^b^	1.3/2.0/18.0/26.7/52.0	*< 0.001*
	Overall image quality	0/12.0/46.7/35.3/6.0^ab^	0/0.7/1.3/28.0/70.0^b^	0.7/2.7/15.3/30.0/51.3	*< 0.001*

*keV* kiloelectron Volt, *MD-iodine* material density iodine

Post hoc pairwise multiple comparisons procedure with the Dunn-Bonferroni test showed a statistically significant (*p* < 0.05) difference between means when compared with 55 keV (^a^), and 70 keV (^b^)

**Table 4:** Inter-reader agreement presented as linearly weighted kappa values for the parameters: lesion conspicuity, pancreatic and surrounding structures, image noise, and overall image quality

Readers, Subgroups	Lesion conspicuity (κ_w_)	*p* value	Image noise (κ_w_)	*p* value	Pancreatic and surrounding structures (κ_w_)	*p* value	Overall image quality (κ_w_)	*p* value
Reader 1 vs. Reader 2	0.274	*< 0.001* ^*^	0.307	*< 0.001* ^*^	-0.067	*0.248*	0.185	*< 0.001* ^*^
Reader 1 vs. Reader 3	0.241	*< 0.001* ^*^	0.368	*< 0.001* ^*^	0.459	*< 0.001* ^*^	0.379	*< 0.001* ^*^
Reader 2 vs. Reader 3	0.183	*< 0.001* ^*^	0.338	*< 0.001* ^*^	-0.143	*0.013* ^*^	0.120	*0.033* ^*^

*κ*_*w*_ weighted kappa

^*^statistically significant

## Discussion

The aim of this study was to compare the image quality between MD-iodine and VMIs at different keV for PCD-CT in the PVP of PDAC at a single tertiary referral center. MD-iodine underperformed compared to 55 keV VMI for both quantitative and qualitative parameters. Our finding of higher CNR on 55 keV compared to MD-iodine contrasts with the results from previous DECT-studies that showed higher CNR on MD-iodine than any VMI keV-level [14, 28]. This could be explained by improved noise properties of PCD at low keV compared to EID in combination with a higher iodine attenuation and contrast due to the inherently increased weight to low photon energies [7, 34]. A phantom PCD-CT study recently demonstrated that PCD-CT had the same noise levels through decreasing keV for both water cavities with and without added iodine, whereas the noise for EID-CT was higher for cavities where iodine was added. Furthermore, the noise increase at low keV was higher for EID-CT compared to PCD-CT for cavities containing iodine [35].

The lack of a significant correlation between CNR and BMI indicates the preservation of CNR at higher BMIs and may be attributed to the inherent design of the PCD, which provides improved noise characteristics and greater signal efficiency. This observation could also suggest that the automatic exposure control of the PCD-CT system is highly effective.

Our result of significantly higher CNR for MD-iodine compared to 70 keV is in line with previous DECT-literature [14, 28]. However, our finding of lowest reader scores for MD-iodine for “lesion conspicuity” and “image noise” contrasts with previous PDAC DECT-studies. One of these studies showed significantly higher reader scores for MD-iodine for “primary tumor visualization” compared to VMI of 50–80 keV, and for “image noise visualization” compared to VMI of 50–70 keV [28]. Another DECT-study showed significantly higher reader scores for MD-iodine for “lesion conspicuity” compared to 52 keV and 70 keV [14]. A third DECT-study showed that there were no significant differences between MD-iodine and 40 keV for “lesion conspicuity” [29].

It has been well established in previous DECT-studies [12–24] and in recent PCD-CT studies [25, 26] that pancreatic carcinoma is better depicted on low energy VMIs. However, both DECT studies [13, 14, 23, 24] and PCD-CT studies [25, 26] have shown that the higher image noise at lower keV-levels can decrease the overall image quality. In this study the 55 keV level for low-keV imaging was chosen because previous DECT-studies have demonstrated favorable image quality outcomes at this keV-level. A DECT-study has shown that VMI at 55 keV in PVP achieved the best tumor conspicuity on subjective analysis when compared to 40 keV, 70 keV, and a polychromatic pancreatic-phase image [23]. In another DECT-study images at 55 keV with a novel monoenergetic reconstruction algorithm received the best subjective image quality scores [24]. Furthermore, a DECT-study demonstrated that the mean CNR-optimized VMI energy level for any pancreatic lesion was 52.5 ± 7.7 keV [17]. A recent PCD-CT study performed a subjective image analysis of PDAC by comparing the tumor delineation and overall image quality between 40 keV and 70 keV. The authors found that “tumor delineation” (corresponding to “lesion conspicuity” in our study) was better at 40 keV but that the overall image quality was better at 70 keV and that a higher number of exams at 40 keV in LAP were rated as “Insufficient” (Score 1) [25]. Another recent PCD-CT study also showed that tumor conspicuity was rated highest on 40 keV but that the overall image quality was rated higher on 50–70 keV [26]. In addition, our initial experience of VMI at 55 keV obtained from our PCD-CT is that the image contrast is already high and that a lower keV-level unnecessarily increases image noise thereby lowering image quality. Altogether, we believe that 55 keV provides an adequate balance between image contrast and noise making it a suitable low-keV imaging energy level candidate for the purpose of image quality comparisons to MD-iodine. The 70 keV VMI reconstruction was chosen because it is considered equivalent to polychromatic 120 kVp images [36], and the mean photon energy at 120 kVp is ∼70 keV [37]. Furthermore, 70 keV is the default reconstruction for abdominal PCD-CT scans at our department. Including more VMI keV-levels in our study would result in more than three CT-series displayed side-by-side on the same layout. This would make simultaneous qualitative evaluation more demanding for the readers and could potentially hamper their judgment.

In this study we chose iodine enhancement in HU-values rather than iodine uptake in mg/ml in the ROI-measurements on MD-iodine to facilitate the comparison between MD-iodine and VMI. Also, this approach mirrors the clinical scenario because the default setting in our clinical PACS-system is to display HU-values in ROI-measurements on MD-iodine. These HU-values correspond to the CT-values of pure iodine. Since this is a PCD-CT system, the values are calculated to correspond to a 70 keV reconstruction. We have also been informed by the vendor of our PCD-CT scanner that the discrepancies between the two methods usually are small in comparison to the other measurement uncertainties. Previous DECT-studies have also chosen to base the CNR calculation on MD-iodine on HU-values [14, 28, 38].

We chose to evaluate MD-iodine a.k.a. “pure iodine images” instead of iodine overlay images because previous DECT-literature on the subject also evaluated MD-iodine [14, 16, 28]. One DECT-study that evaluated both MD-iodine and iodine overlay images for pancreatic lesions of any kind concluded that there was no statistically significant difference of subjective ratings for lesion conspicuity and reader confidence between the two image reconstructions [29].

Regarding inter-reader agreement, κ_w_ values were lower between R2 and the other readers than those between R1 and R3 for the parameters “image noise”, “structures” and “overall image quality” (Table 4). A closer analysis of the readers’ scores (Supplementary Table 2, and Supplementary Fig. 1) revealed that R2 gave higher scores to MD-iodine, compared to the other readers. Notably, R2 graded MD-iodine significantly higher compared to 70 keV for the parameter “structures”. The underlying reason for this discrepancy is unclear but may be attributed to R2, one of the more experienced abdominal radiologists, having greater familiarity with MD-iodine images. An additional noteworthy observation is that R3, the least experienced of the three readers, consistently assigned the highest scores to 70 keV across all parameters (Supplementary Table 2). In contrast, the more experienced readers, R1 and R2, assigned the highest scores to 70 keV only for the parameter “image noise”. This may be explained by R3’s limited experience with reconstructions other than 70 keV, which closely resembles 120 kVp images. Further investigation, involving a greater number of less experienced readers, is warranted to validate these findings in future studies.

MD-iodine demonstrated a discrepancy between quantitative and qualitative image quality. It showed significantly higher CNR compared to 70 keV but received significantly lower qualitative scores for “lesion conspicuity”. A probable major contributing factor to this is that the readers graded “image noise” as significantly highest (lowest scores) on MD-iodine.

The main limitation in this study was the retrospective design at a single tertiary referral center. Second, we did not investigate the diagnostic performance of the different image reconstructions directly. Third, the number of cases is relatively small; however, this is expected given the rarity of the disease. Fourth, the entire cohort included both primary and recurrent tumors, affecting the homogeneity of our results. Fifth, 27 out of the 50 PCD-CT examinations involved patients undergoing chemotherapy. We included these patients because adjuvant chemotherapy is routinely administered in most cases. Sixth, we did not fully leverage the high spatial resolution capabilities of the PCD-CT scanner because images were reconstructed with a 3 mm slice thickness. The rationale behind this choice is based on our initial experience that thinner slices result in a significant increase in noise on MD-iodine, an issue that has also been highlighted in recent literature [27]. Additionally, we chose to adhere to a slice thickness that our abdominal radiologists are well accustomed to, as 3 mm with a 1.5 mm increment is standard in our routine clinical abdominal CT protocol. Moreover, we sought to align with existing literature on the diagnostic potential of MD-iodine in pancreatic imaging, where a slice thickness of 2–2.5 mm has been employed [14, 28, 29].

In conclusion, despite demonstrating a higher CNR, MD-iodine had significantly lower image quality scores compared to 70 keV. 55 keV performed better overall than MD-iodine for both quantitative and qualitative image quality parameters and had no non-diagnostic scans.

## Electronic supplementary material

Below is the link to the electronic supplementary material.

Supplementary Material 1: Stacked bar chart showing the scores for each specific reader as percentages for MD-iodine, 55 keV, and 70 keV. The columns represent the qualitative image quality parameters (“lesion conspicuity”, “image noise”, pancreatic and surrounding structures (“structures”), and “overall image quality”) and the rows represent the different readers (Reader 1–3).

Supplementary Material 2

Supplementary Material 3

## Data Availability

No datasets were generated or analysed during the current study.
