# Supplementary figures and images for: Photon-counting detector computed tomography: iodine density versus virtual monoenergetic imaging of pancreatic ductal adenocarcinoma
Source: Abdom Radiol (NY). 2024 Sep 26;50(4):1720–30. doi: 10.1007/s00261-024-04605-0 (PMC11946985; doi:10.1007/s00261-024-04605-0)

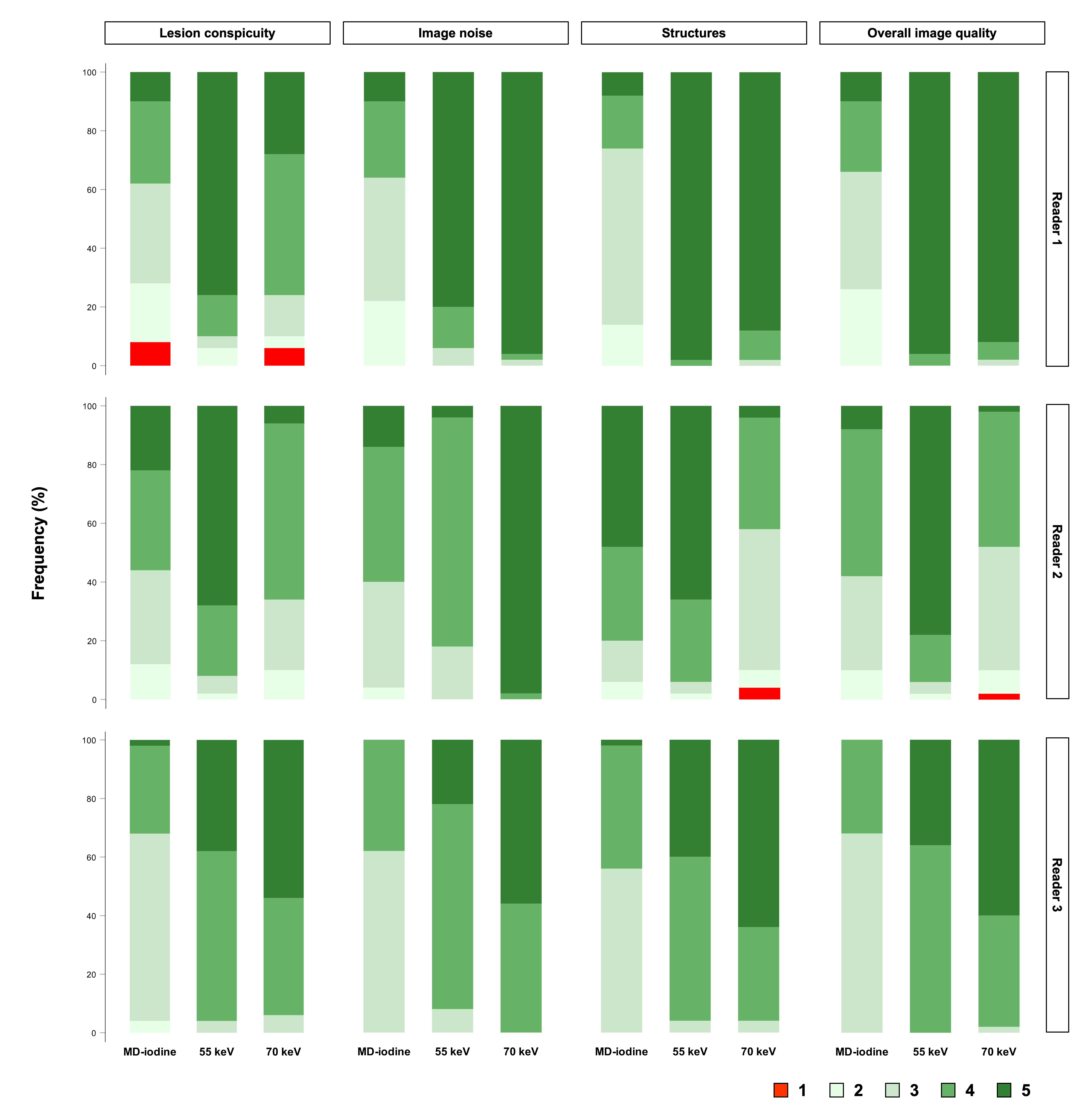

Supplement: Supplementary file 1 — Supplementary Material 1: Stacked bar chart showing the scores for each specific reader as percentages for MD-iodine, 55 keV, and 70 keV. The columns represent the qualitative image quality parameters (“lesion conspicuity”, “image noise”, pancreatic and surrounding structures (“structures”), and “overall image quality”) and the rows represent the different readers (Reader 1–3). [file 261_2024_4605_MOESM1_ESM.png]
