# Supplementary material for: Photon-counting detector computed tomography: iodine density versus virtual monoenergetic imaging of pancreatic ductal adenocarcinoma
Source: Abdom Radiol (NY). 2024 Sep 26;50(4):1720–30. doi: 10.1007/s00261-024-04605-0 (PMC11946985; doi:10.1007/s00261-024-04605-0)
Supplement: Supplementary file 2 — Supplementary Material 2 [file 261_2024_4605_MOESM2_ESM.docx]

**Supplementary Table 1.**  Bonferroni adjusted post hoc pairwise p-values from the qualitative image quality analysis, for all reconstruction type pairs

| **Parameters, Subgroups** | | **MD-iodine vs. 55 keV** | **55 keV vs.**  **70 keV** | **MD-iodine vs. 70 keV** |
| --- | --- | --- | --- | --- |
|  | Lesion conspicuity | < 0.001 | < 0.001 | < 0.001 |
|  | Image noise | < 0.001 | < 0.001 | < 0.001 |
|  | Pancreatic and surrounding structures | < 0.001 | 0.003 | < 0.001 |
|  | Overall image quality | < 0.001 | 0.001 | < 0.001 |

*keV* kiloelectron volts, *MD-iodine* material density iodine
