# Supplementary material for: Photon-counting detector computed tomography: iodine density versus virtual monoenergetic imaging of pancreatic ductal adenocarcinoma
Source: Abdom Radiol (NY). 2024 Sep 26;50(4):1720–30. doi: 10.1007/s00261-024-04605-0 (PMC11946985; doi:10.1007/s00261-024-04605-0)
Supplement: Supplementary file 3 — Supplementary Material 3 [file 261_2024_4605_MOESM3_ESM.docx]

**Supplementary Table 2.** Comparison of qualitative image quality scores for the abdominal portal venous phase CT series between MD-iodine, 55 keV, and 70 keV

| **Reader, Parameters, Subgroups (Distribution of scores 1/2/3/4/5 given as percentages)** | | **MD-iodine** | **55 keV** | **70 keV** | ***p* value** |
| --- | --- | --- | --- | --- | --- |
| **Reader 1** | |  |  |  |  |
|  | Lesion conspicuity | 8.0/20.0/34.0/28.0/10.0^ab^ | 0/6.0/4.0/14.0/76.0^b^ | 6.0/4.0/14.0/48.0/28.0 | *< 0.001* |
|  | Image noise | 0/22.0/42.0/26.0/10.0^ab^ | 0/0/6.0/14.0/80.0 | 0/0/2.0/2.0/96.0 | *< 0.001* |
|  | Pancreatic and surrounding structures | 0/14.0/60.0/18.0/8.0^ab^ | 0/0/0/2.0/98.0 | 0/0/2.0/10.0/88.0 | *< 0.001* |
|  | Overall image quality | 0/26.0/40.0/24.0/10.0^ab^ | 0/0/0/4.0/96.0 | 0/0/2.0/6.0/92.0 | *< 0.001* |
| **Reader 2** | |  |  |  |  |
|  | Lesion conspicuity | 0/12.0/32.0/34.0/22.0^a^ | 0/2.0/6.0/24.0/68.0^b^ | 0/10.0/24.0/60.0/6.0 | *< 0.001* |
|  | Image noise | 0/4.0/36.0/46.0/14.0^b^ | 0/0/18.0/78.0/4.0^b^ | 0/0/0/2.0/98.0 | *< 0.001* |
|  | Pancreatic and surrounding structures | 0/6.0/14.0/32.0/48.0^b^ | 0/2.0/4.0/28.0/66.0^b^ | 4.0/6.0/48.0/38.0/4.0 | *< 0.001* |
|  | Overall image quality | 0/10.0/32.0/50.0/8.0^a^ | 0/2.0/4.0/16.0/78.0^b^ | 2.0/8.0/42.0/46.0/2.0 | *< 0.001* |
| **Reader 3** | |  |  |  |  |
|  | Lesion conspicuity | 0/4.0/64.0/30.0/2.0^ab^ | 0/0/4.0/58.0/38.0 | 0/0/6.0/40.0/54.0 | *< 0.001* |
|  | Image noise | 0/0/62.0/38.0/0^ab^ | 0/0/8.0/70.0/22.0^b^ | 0/0/0/44.0/56.0 | *< 0.001* |
|  | Pancreatic and surrounding structures | 0/0/56.0/42.0/2.0^ab^ | 0/0/4.0/56.0/40.0 | 0/0/4.0/32.0/64.0 | *< 0.001* |
|  | Overall image quality | 0/0/68.0/32.0/0^ab^ | 0/0/0/64.0/36.0 | 0/0/2.0/38.0/60.0 | *< 0.001* |

*keV* kiloelectron Volt, *MD-iodine* material density iodine

Post hoc pairwise multiple comparisons procedure with the Dunn-Bonferroni test showed a statistically significant (*p* <0.05) difference between means when compared with 55 keV (^a^), and 70 keV (^b^)
